# Solvent-Free Lipase-Catalyzed Synthesis of Diacylgycerols as Low-Calorie Food Ingredients

**DOI:** 10.3389/fbioe.2016.00006

**Published:** 2016-02-10

**Authors:** Luis Vázquez, Noemí González, Guillermo Reglero, Carlos Torres

**Affiliations:** ^1^Departamento de Producción y Caracterización de Nuevos Alimentos, Instituto de Investigación en Ciencias de la Alimentación (CSIC-UAM), Universidad Autónoma de Madrid, Madrid, Spain; ^2^IMDEA-Food Institute, CEI (UAM-CSIC), Madrid, Spain

**Keywords:** diacylglycerol, fatty acid ethyl ester, lipase, medium-chain fatty acid, transesterification, solvent-free

## Abstract

Problems derived from obesity and overweight have recently promoted the development of fat substitutes and other low-calorie foods. On the one hand, fats with short- and medium-chain fatty acids are a source of quick energy, easily hydrolyzable and hardly stored as fat. Furthermore, 1,3-diacylglycerols are not hydrolyzed to 2-monoacylglycerols in the gastrointestinal tract, reducing the formation of chylomicron and lowers the serum level of triacylglycerols by decreasing its resynthesis in the enterocyte. In this work, these two effects were combined to synthesize short- and medium-chain 1,3-diacylglycerols, leading to a product with great potential as for their low-calorie properties. Lipase-catalyzed transesterification reactions were performed between short- and medium-chain fatty acid ethyl esters and glycerol. Different variables were investigated, such as the type of biocatalyst, the molar ratio FAEE:glycerol, the adsorption of glycerol on silica gel, or the addition of lecithin. Best reaction conditions were evaluated considering the percentage of 1,3-DAG produced and the reaction rate. Except Novozym 435 (*Candida antarctica*), other lipases required the adsorption of glycerol on silica gel to form acylglycerols. Lipases that gave the best results with adsorption were Novozym 435 and Lipozyme RM IM (*Rhizomucor miehei*) with 52 and 60.7% DAG at 32 h, respectively. Because of its specificity for *sn*-1 and *sn*-3 positions, lipases leading to a higher proportion of 1,3-DAG vs. 1,2-DAG were Lipozyme RM IM (39.8 and 20.9%, respectively) and Lipase PLG (*Alcaligenes* sp.) (35.9 and 19.3%, respectively). By adding 1% (w/w) of lecithin to the reaction with Novozym 435 and raw glycerol, the reaction rate was considerably increased from 41.7 to 52.8% DAG at 24 h.

## Introduction

Obesity and overweight are becoming a major concern in industrialized countries. These disorders normally are related to a high fat intake in diet. In addition, many health disorders including heart disease, diabetes, and cancer are associated with obesity. Many public health agencies and professional health organizations have recommended to reduce fat consumption, especially saturated fat. To minimize risks, consumers are controlling mainly the total amount of fat in their diet (Thompson et al., 1999; Huang, 2007). In response to consumer demands for low-calorie fats, a wide array of fat replacers and other lower-calorie dietary items to enhance health have been developed (Shahidi and Senanayake, 2006). These fat replacers provide similar mouthfeel texture or flavor to a food product as normal fat. Fat can be replaced by reformulating the foods with lipid-, protein-, or carbohydrate-based ingredients, individually or in combination (Akoh, 1998).

Fat-based fat replacers work in several ways. Some types of fat-based replacers change normal fat properties by binding fatty acids to sugars, such as olestra or sorbestrin. These cannot be absorbed by the body and yield no energy value. Other fat replacers based in fatty compounds are triacylglycerols comprised of combinations of short and saturated long-chain fatty acids, which supply fewer calories and can be only partially digested and absorbed. Salatrim (Benefat) and Caprenin are commercial examples of TAG contributing about 5 kcal/g, while energy intake of conventional fats is 9 kcal/g.

Medium-chain triacylglycerols (MCT) have been the subject of much attention due to their properties as low-calorie fats. In MCT, all three positions of the glycerol molecule are esterified with medium-chain fatty acids (MCFA). The main source of MCFA (C6–C12) is butterfat, with a small contribution from coconut and palm kernel oils. Normally, MCFA are minor dietary components in the diet. However, MCFA can provide a rapid source of energy that is readily absorbed and easily digested. As an example, Liao et al. (1984) found in a study *in vivo* in animals that MCT were hydrolyzed by lipase five to eight times more than the long chain TAG. Short- and medium-chain fatty acids are hardly incorporated into chylomicrons in the enterocyte (Bach and Babayan, 1982), and they are mainly transported directly to the liver for use as an energy source. Hence, compared to long-chain fatty acids, MCFA are not re-esterified so easily to TAG and hence are not stored as body fat (Geliebter et al., 1983). For these reasons, MCT can constitute a part of the fat blend of liquid diet formulas for patients with impaired digestion or diverse medical conditions requiring fluid restriction, such as AIDS, cystic fibrosis, postoperative cancer patients, multiple traumas, burn injury, respiratory distress, hepatic, or renal disease (Huang, 2007).

Furthermore, some types of structured diacylglycerols are used as fat-based fat replacers. These molecules are comprised of fatty acids esterified at the *sn-*1 and *sn-*3 positions on the glycerol backbone. 1,3-DAG is metabolized differently than triacylglycerols (TAG) because they are not hydrolyzed to 2-MAG in the intestine. The *sn-*1- or *sn-*3-monoacylglycerols produced in the intestine from 1,3-DAG hydrolysis are poorly reassembled into chylomicrons (Hara et al., 1993; Murata et al., 1997). Some of the hydrolysis products reach the liver, where they are used for energy purposes rather than as a fat storage material in the adipose tissue. In addition, 1,3-DAG reduces the formation of chylomicron and lowers the serum level of triacylglycerol by decreasing the resynthesis of TAG in the intestine. Results from clinical trials in Japan and the United States have shown that 1,3-DAG oil substituted for conventional vegetable oil can reduce body weight and fat mass (Nagao et al., 2000; Maki et al., 2002) and prevent obesity (Flickinger and Matsuo, 2003).

There are various methods for the modification of oils and fats as hydrogenation, fractionation, blending, interesterification, esterification, and even biotechnology. The preferred method is interesterification, either chemical or enzymatic. Although chemical interesterification is a technology widely used in the industry, it generally can only provide TAG with a random distribution. For example, Salatrim and Caprenin are industrially produced by chemical interesterification between short- and medium-chain TAG and TAG comprising saturated long-chain fatty acids from hydrogenated vegetable oils.

In this field, 1,3-DAG can be synthesized from oils and fats by using glycerolysis reactions, temperatures above 200°C and alkaline catalysts. However, low levels of yield and purity have been attained using this methodology, which makes it not suitable for industrial production (Sontang, 1984). On the other hand, enzymatic interesterification with sn-1,3-specific lipases has also been used in industry for the production of low-calorie fats, cocoa butter substitutes, infant formulas, and other structured lipids. The use of lipases as biocatalysts in the production of structured lipids has several benefits (Xu, 2000). The most important merits are their efficacy under mild reaction conditions, utility in “natural” reaction systems and products, reduced environmental pollution, availability of lipases from a wide range of sources, ability to improve lipases by genetic engineering, and in special situations, the use of lipases for the production of particular biomolecules. Besides, utilization of immobilized enzymes for production of fat replacers has also become very interesting, since it makes insoluble forms, which retain their catalytic activity and which can be reused repeatedly (Brena and Batista-Viera, 2006).

Numerous research groups have studied the production of 1,3-DAG *via* enzymatic reactions in the presence of lipases (Yamane et al., 1994; Kwon et al., 1995; Xu et al., 1999; Watanabe et al., 2003; Vu et al., 2008). Yamane et al. (1994) studied the enzymatic glycerolysis of beef tallow to obtain 1,3-DAG. Plou et al. (1996) reported a method for producing 1,3-DAG by hydrolysis of triolein, but low yield and a large amount of MAG was obtained in the reaction.

Berger et al. (1992a) reported on the production of 1,3-DAG by esterification using FA and glycerol in an organic solvent containing trace amount of water. As an alternative to using organic solvent, Rosu et al. (1999) synthesized 1,3-DAG by esterification of FA and glycerol in a solvent-free system using 1,3-regioselective lipases (*Rhizomucor miehei* lipase) with the simultaneous removal of water. The authors utilized caprylic acid as a source of FA achieving a 1,3-DAG yield of 84.6% with a purity of 96%. Other authors have optimized the reaction conditions for production of DAG by esterification of glycerol and FA using Lipozyme RM IM (*R. miehei* lipase) (Watanabe et al., 2003).

Xu et al. studied different parameters affecting diacylglycerol formation during the production of specific-structured lipids by lipase-catalyzed interesterification. These authors evaluated the influence of water content, reaction temperature, enzyme load, substrate molar ratio (oil/capric acid), acyl migration, and reaction time on the formation of DAG in batch reactors (Xu et al., 1999).

In this research field, the group of Akoh has successfully developed the synthesis of structured lipids containing short- and medium-chain fatty acids among others to produce functional dietary lipid molecules (Akoh and Yee, 1997; Fomuso and Akoh, 1997; Vu et al., 2008). Fureby et al. investigated the more efficient acyl group donor for the formation of DAG and low formation of TAG. They compared the behavior of TAG, free fatty acid, and capric acid ethyl ester (C10:0), the latter being the one that reached the greater yield (Fureby et al., 1996).

Watanabe et al. (2003) found that using an immobilized enzyme, the esterification of glycerol with fatty acids occurred only when the enzyme contact first with the oily phase and not with the glycerol. Xu et al. (2011) explained this behavior by several mechanisms. One hypothesis is the formation of a coating layer of glycerol that avoids the diffusion of substrates to the enzyme. Another possible mechanism was a decreased water activity based on the hygroscopic nature of glycerol. Accordingly, several studies have described the use of silica gel to adsorb glycerol and overcome these problems (Dossat et al., 1999; Yesiloglu, 2004). Hence, Castillo et al. (1997) performed esterification reactions with oleic acid in hexane and found that the conversion was remarkably increased by adsorption of glycerol in silica gel and duolite. According to this study, Berger et al. (1992a) and Kwon et al. (1995) also reported that the glycerol adsorption on a solid support and the nature of the adsorbent agent are critical to the success of the esterification reaction.

Selmi et al. (1997) concluded that the presence of glycerol adsorbed on silica gel enhances the initial rate of synthesis of di- and tricaprylin. Due to the strong affinity for glycerol, the silica gel acts as a “reservoir” of glycerol and plays a protective role for the immobilized enzyme, preventing the polar substrate (glycerol) is adsorbed on the enzyme support (Stevenson et al., 1994). Another possible reason is that the adsorption of glycerol on silica gel glycerol facilitates the contact between the enzyme, glycerol (hydrophilic), and caprylic acid (hydrophobic).

However, currently the transesterification reactions between glycerol and fatty acid esters of short and medium chain for producing acylglycerols in solvent-free systems have not been investigated in depth. The reason may be due to the low miscibility of substrates involved and the problems associated to this procedure, such as the enzyme coating by glycerol. The aim of this work has been the synthesis of 1,3-DAG comprising short- and medium-chain fatty acids *via* lipase-catalyzed reactions and without employing organic solvents.

## Materials and Methods

### Materials

The short- and medium-chain fatty acid ethyl esters (FAEE) were obtained utilizing a previous methodology described by Vázquez and Akoh (2010). In that work, fractionation of FAEE produced by ethanolysis of a blend of coconut oil and milk fat was carried out by molecular distillation. Table 1 shows the FAEE composition.

**Table 1:** **Short- and medium-chain FAEE obtained by molecular distillation from an ethanolysis product of a blend of coconut oil and milk fat (Vázquez and Akoh, 2010)**.

Fatty acid ethyl ester	Composition (% wt)
Butyric acid (C4:0)	8.4
Caproic acid (C6:0)	8.9
Caprylic acid (C8:0)	29.9
Capric acid (C10:0)	18.1
Undecanoic acid (C11:0)	0.6
Lauric acid (C12:0)	29.4
Myristic acid (C14:0)	3.4
Myristoleic acid (C14:1)	0.1
Pentadecanoic acid (C15:0)	0.1
Palmitic acid (C16:0)	0.9
Stearic acid (C18:0)	0.1
Oleic acid (C18:1 n9)	0.2
Linoleic acid (C18:2 n6)	0.1

Glycerol (99.5%) and silica gel 60 (230-400 mesh) were obtained from Scharlau (Barcelona, Spain); *n*-hexane (95%), metil *tert*-butil ether (HPLC grade), and isopropanol (HPLC grade) from Lab Scan (Gliwice, Poland); absolute ethanol and formic acid (98%) from Panreac Quimica (Barcelona, Spain); isooctane (HPLC grade) from Carlo Erba; and egg lecithin (96% phosphatidylcholine) from Lipoid (Ludwigshafen, Germany).

Butyric (C4:0), caprylic (C8:0), lauric (12:0), and oleic (C18:1) acid ethyl esters used as external standards in GC, monoacylglycerols: 1-lauroyl-*rac*-glycerol (≥99%) and 1-octanoyl-*rac*-glycerol (≥99%) used as external standards in HPLC, and squalane (99%) used as internal standard, were purchased from Sigma Chemical Co. (St. Louis, MO, USA).

As biocatalysts, the following immobilized commercial lipases were used: Novozym^®^ 435 (*Candida antarctica* B), Lipozyme^®^ TL IM (*Thermomyces lanuginosa*), and Lipozyme^®^ RM IM (*R. miehei*) obtained from Novozymes A/S (Bagsvaerd, Denmark); Lipase SL (*Burkholderia cepacia*), Lipase TL (*Pseudomonas stutzeri*), and Lipase PLG and QLC (*Alcaligenes* sp.) obtained from Meito Sangyo Co. (Tokyo, Japan).

### Methods

#### Lipase-Catalyzed Transesterification Reactions with FAEE and Glycerol

Synthesis of short- and medium-chain DAG was performed by enzymatic transesterification reactions with FAEE and glycerol. In this study, several variables were investigated, such as the type of biocatalyst, form of glycerol, substrate molar ratio, temperature, amount of biocatalyst, and vacuum.

Hence, two screenings with the immobilized commercial lipases previously mentioned in Section “Materials” based on the utilization of two different forms of glycerol were evaluated: raw glycerol (liquid) and glycerol adsorbed on silica gel.

Regarding the molar ratio, in a preliminary study, two conditions were evaluated: (2:1) and (3:1) (FAEE:glycerol) for Novozym 435 with raw glycerol. For the rest of experiments, (2:1) (FAEE:glycerol) molar ratio was selected. All reactions were performed at 65°C. This relatively high temperature was selected to increase the reaction rate and improve the miscibility of FAEE and glycerol. The amount of lipase in all reactions was 10% (w/w of the total reaction mixture). All the reactions were carried out under vacuum of 200 mbar. This vacuum was selected in order to volatilize the ethanol produced by reaction. Elimination of ethanol from the reaction mixture shifts the equilibrium toward the production of DAG. Moderate reduced pressure was utilized to avoid the partial elimination of the most volatile FAEE in the reaction mixture. Hence, 200 mbar was selected taking into account the evaporation curves at different vacuums of ethanol and ethyl butyrate (the most volatile FAEE present in the mixture) and its behavior at 65°C.

The addition of 1% wt lecithin was also studied in two experiments: with Novozym 435 and Lipozyme RM IM, both with raw glycerol. The aim of this study was to improve the miscibility of glycerol and FAEE at the first reaction times.

In a transesterification reaction, FAEE (4 g) and glycerol (1 g) were mixed in a 100-mL flask with a molar ratio of 2:1, FAEE:glycerol. In reactions with glycerol adsorbed on silica gel, 2.5 g of silica gel:glycerol mixture (1.5:1, w/w) were added to FAEE (4 g). About 1% (w/w) of lecithin was also added when required. The flask was placed in a thermostatically controlled bath (with water or thermal fluid) at 65°C and magnetic stirring. When 65°C temperature was reached, 0.5 g of lipase (10%, w/w) was added to the medium and vacuum at 200 mbar (Büchi Vacuum Controller B-721) was coupled to the system.

Samples of 80 μL were withdrawn periodically in a 2-mL *eppendorf* vial. Then, 15 μL squalane were added as internal standard and the mixture was dissolved in 1.5 mL hexane:ethanol 80:20 (v/v). This solution was centrifuged at 14,500 rpm in a micro-centrifuge Scanspeed Mini for 2 min in order to separate the lipase from the reaction medium. The supernatant was subsequently analyzed by GC and HPLC.

#### Glycerol Immobilization on Silica Gel

Silica gel (1 g) was mixed with glycerol (1 g) in a 30-mL flask. This mixture was manually homogenized with spatula. When the mixture was almost homogeneous, another 0.5 g silica gel was added. The final mixture [1.5:1 (w/w) silica gel:glycerol] was then homogenized with magnetic stirring overnight.

#### GC Analysis

Fatty acid ethyl esters were analyzed by GC. About 1 μL of the diluted sample was injected into an Agilent gas chromatograph (6890N Network GC System) coupled to an autosampler (Agilent 7683B). The capillary column was an HP-88 (30 m, 0.25 mm i.d.). A 20:1 split ratio was used. The injector and detector temperatures were 220 and 250°C, respectively. The temperature program started at 100°C, rising to 180°C at 20°C/min, followed by heating from 180 to 220°C at 15°C/min. The final temperature (220°C) was held for 10 min.

**Figure 1:**
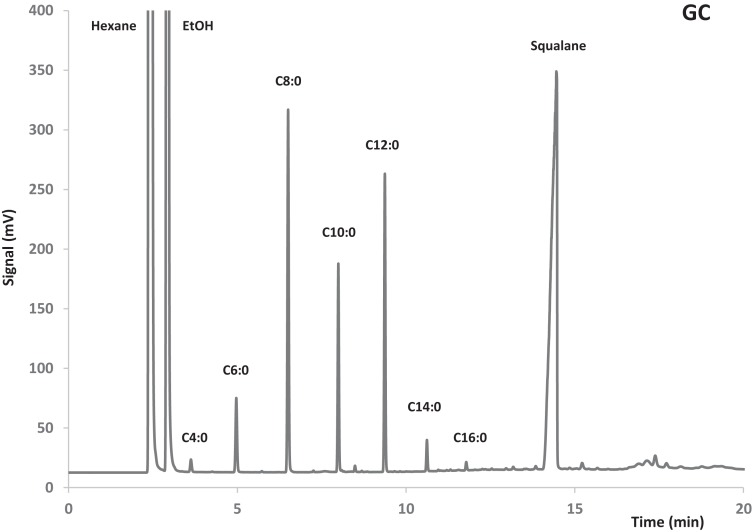
**Example of GC chromatogram: FAEE from a reaction product obtained with Lipozyme RM IM**.

Quantification was effected *via* external standard of butyric, caprylic, lauric, and oleic acid ethyl esters. Squalane was used as internal standard to determine the total amount of sample injected in the gas chromatograph. An example of GC chromatogram is shown in Figure 1.

#### HPLC Analysis of Acylglycerols

The MAG, DAG, and TAG composition was analyzed on a Agilent poroshell 120 (2.7 μm, 100 mm × 4.6 mm) coupled to an Agilent 1200 Series HPLC (Santa Clara, CA, USA) containing a thermostated column compartment (35°C), a quaternary pump, an autosampler, a vacuum degasser, and evaporative light scattering detector (ELSD). Conditions of the ELSD were 3.5 bar, 35°C, and gain 3. The flow rate was 2 mL/min and the injection volume was 1 μL. A split valve was used after the column, and only 50% of the mobile phase was directed through the detector. Table 2 shows the mobile phase utilized, previously reported by Torres et al. (2005).

**Table 2:** **Mobile phase used for the HPLC analysis**.

Min	Flow (mL/min)	Solvent A (%)	Solvent B (%)	Solvent C (%)
00.00	1	100	0	0
02.01	2	97	3	0
05.00	2	95	5	0
08.00	2	82	18	0
12.00	2	50	50	0
12.01	2	46	50	4
23.40	2	50	50	0
28.00	2	100	0	0
29.00	1	100	0	0
32.00	1	100	0	0

*Solvent A: isooctane; solvent B: isooctane/methyl *tert*-butyl ether 50/50 (v/v) with 0.02% formic acid; solvent C: methyl *tert*-butyl ether/isopropanol 50/50 (v/v)*.

**Figure 2:**
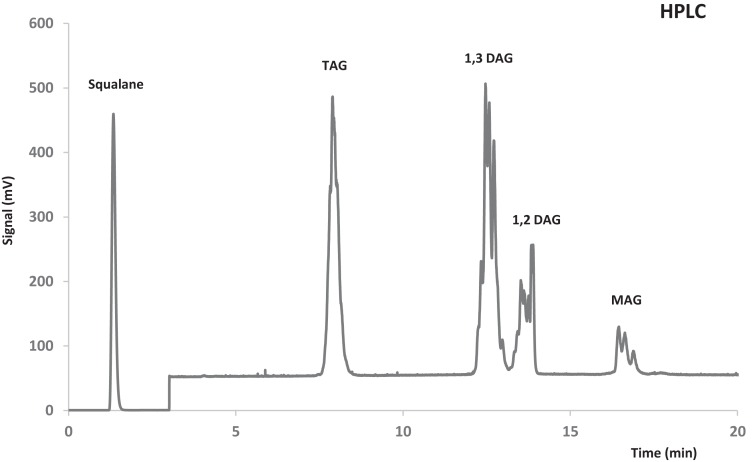
**Example of HPLC chromatogram: reaction product obtained with Novozym 435**.

A mixture comprised of 1-lauroyl-rac-glycerol and 1-octanoyl-rac-glycerol was used as external standard for quantification of MAG. Products obtained by semipreparative HPLC were used as external standard for quantification of DAG and TAG. For this purpose, intermediates of the transesterification reaction were fractionated in a preparative HPLC AV-3740 Kromasil Sil 5 μm (2500 mm × 10 mm) column (Análisis Vínicos, Tomelloso, España). An example of HPLC chromatogram is shown in Figure 2.

#### Glycerol Analysis

The remaining glycerol in products obtained at optimal reactions was also determined. This analysis was performed according to the spectrophotometric method described by Bondioli and Della Bella (2005). Hence, the sample with glycerol was homogenized at 50°C and diluted in isopropanol at 10 mg/mL. Several aliquots were prepared from this solution. About 1 mL of A reagent (65 mg NaIO_4_, 90 mL water, 10 mL acetic acid, and 7.7 g ammonium acetate) was added to each aliquot, and this mixture was held at 50°C for 5 min. Subsequently, 2.5 mL of B reagent (1 mL acetylacetone in 99 mL isopropanol) were added. This mixture was held at 50°C for 20 min. Finally, the absorbance was measured at 410 nm. For the quantification of glycerol, a standard curve with different concentrations of glycerol was prepared.

## Results and Discussion

Transesterification reactions between FAEE of short- and medium-chain FAEE and glycerol were studied in order to produce low-calorie DAG. Some possible problems associated to these reactions are the low miscibility between the substrates, and the possible coating of the active site of the lipase by glycerol (Xu et al., 2011).

According to this, a first screening of different lipases was carried out using raw glycerol as substrate. The reaction conditions are detailed in Section “Methods.” Table 3 shows the % FAEE (unesterified with glycerol) in the lipid phase of the reaction medium at 24 h. It should to be noted that no hydrolysis was observed in these reactions because free fatty acids were not detected. For that reason, the % FAEE was used to evaluate the conversion rate into acylglycerols for the different lipases, since the % FAEE is inversely proportional to the formation of acylglycerols.

**Table 3:** **% FAEE in the product mixture after 24 h of transesterification reaction**.

Lipase	FAEE (% wt)
Lipase QLC	100
Lipozyme TL IM	99.7
Lipase PLG	99.9
Novozym 435	24.3
Lipozyme RM IM	85.2
Lipase SL	70.9

This table shows that Novozym 435 lipase produced the highest FAEE conversion. Almost negligible conversion of FAEE was observed in the presence of the other lipases tested that could be attributed to an enzyme coating by glycerol.

### FAEE:Glycerol Molar Ratio Study

**Figure 3:**
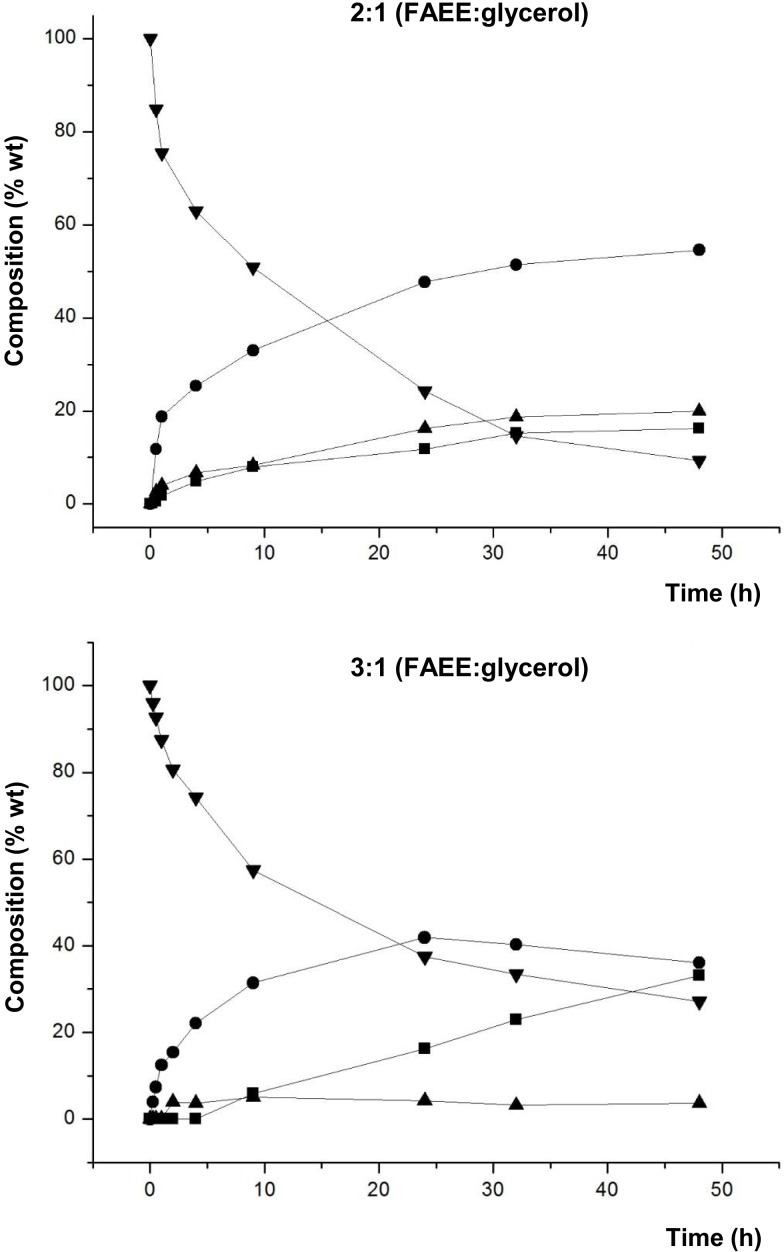
**Composition (% w/w) of FAEE (▼), TAG (■), DAG (●), and MAG (▲) at different reaction times of transesterification between FAEE and raw glycerol with Novozym 435 (*Candida antarctica*)**. Substrate molar ratio 2:1 (FAEE:glycerol): 4 g FAEE and 1 g glycerol. Substrate molar ratio 3:1 (FAEE:glycerol): 6.5 g FAEE and 1 g glycerol.

Subsequently, Novozym 435 was used to evaluate the effect of the substrate molar ratio. Figure 3 shows the composition in % by weight (lipid phase without glycerol) of the aliquots obtained at different reaction times, using molar ratios 2:1 and 3:1 (FAEE:glycerol), respectively. The rest of reaction conditions were identical to those used in the previous screening.

Figure 3 shows that formation of DAG was higher at molar ratio 2:1 (FAEE:glycerol). Specifically, the highest percentage (54.6% DAG) at 2:1 (FAEE:glycerol) ratio was reached after 48 h, whereas the maximum for 3:1 (FAEE:glycerol) ratio was 41.9% DAG after 24 h. Besides, as expected, the remaining % FAEE in the product mixture was lower with the ratio 2:1 compared to 3:1 (FAEE:glycerol). For this reason, the molar ratio 2:1 (FAEE:glycerol) was selected for further studies.

### Lipase-Catalyzed Reactions to Synthesize DAG with Glycerol Adsorbed on Silica Gel

A second screening was performed by using glycerol adsorbed on silica as starting substrate. The objective of these experiments was to avoid the inhibition by coating of the active site of the lipase by glycerol. The reaction conditions were detailed in Section “Methods.”

**Figure 4:**
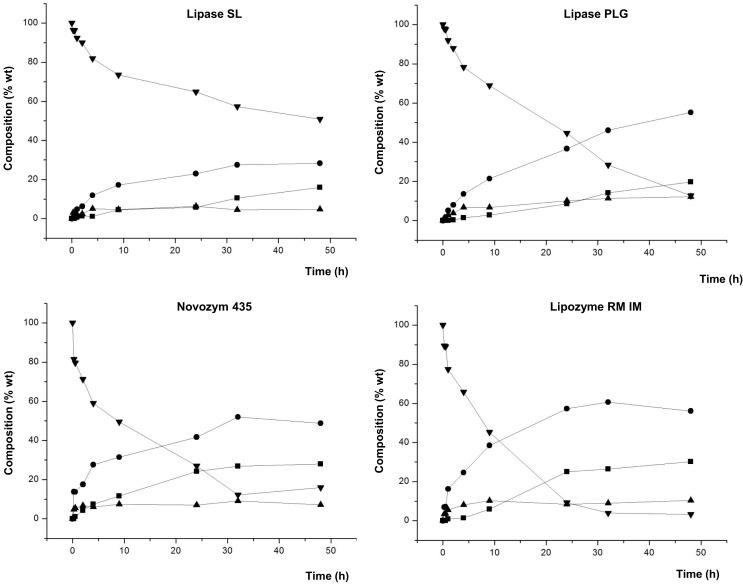
**Composition (% w/w) of FAEE (▼), TAG (■), DAG (●), and MAG (▲) at different reaction times of transesterification between FAEE and glycerol adsorbed on silica gel with Lipase SL (*Burkholderia cepacia*), Lipase PLG (*Alcaligenes* sp.), Novozym 435 (*Candida antarctica*), and Lipozyme RM IM (*Rhizomucor miehei*)**.

Figure 4 shows the composition of the product mixture (glycerol free) at different reaction times for the different lipases studied.

Lipozyme TL IM and Lipase TL led to a low conversion into acylglycerols (data not shown in the figure). For these lipases, the highest values for DAG were 13.7 and 25.2%, respectively, whereas Lipase SL gave 28.3% DAG. On the contrary, as observed in Figure 4, Lipase PLG gave a maximum of 55.2% for DAG at 48 h, with a low amount of FAEE (12.8%) in the final product. Likewise, 52 and 60.7% DAG were attained at 32 h with Novozym 435 and Lipozyme RM IM, respectively. These three lipases led to the highest composition of DAG in the final product.

In contrast to the study with raw glycerol (unadsorbed) (see Table 3), a remarkable difference is observed in this screening, since all the lipases led to formation of acylglycerols, in varying degrees, and the conversion was significantly most effective when glycerol was adsorbed on silica. These results reinforce the conclusions of studies by Castillo et al. (1997), Berger et al. (1992b), Kwon et al. (1995), and Selmi et al. (1997), wherein the adsorption of glycerol on silica gel significantly enhanced the effectiveness of esterification reactions. Also, it should be noted that in this study, a solvent-free reaction medium was used, following the development of a Green Chemistry, and in contrast to the studies mentioned above, with the exception of performed by Castillo et al.

Moreover, whereas with Lipozyme TL IM, the reaction did not progress after 4–9 h (data not shown in the figure), with both Lipase TL and Lipase SL, the maximum conversion was attained after 24–32 h. Another important aspect to discuss from Figure 4 is the different reaction rates by the lipases studied. Hence, comparing the three lipases which led to a higher production of total DAG (Novozym 435 (52%), Lipozyme RM IM (60.7%), and Lipase PLG (55.2%) (Figure 4) shows that the first two gave the highest DAG at 32 h, while the latter required 48 h. Hence, at 32 h, Lipase PLG formed 46.1% DAG. Thus, we can conclude that Novozym 435 and Lipozyme RM IM are enzymes that besides giving rise to a greater amount of DAG, resulted in a higher reaction rate.

In previous works, other authors achieved higher conversions into 1,3-diacylglycerols by direct esterification in solvent-free systems and Lipozyme RM IM as catalyst. These authors used oleic and linoleic acid (Watanabe et al., 2003) and mixtures including MCFA (Rosu et al., 1999) in their free fatty acid form as acyl donors. The differences with the present study can be explained by the different form of the acyl donor employed. Thus, in our work, more volatile FAEE were used instead of free fatty acids with less volatility. Besides, this FAEE fraction consisted only of short- and medium-chain compounds, so its volatility was increased. For that reason, in our work, the reduced pressure had to be adjusted to 200 mbar in order to minimize the partial elimination of the most volatile FAEE, whereas in other works, a decreased vacuum of 4 mbar was possible. The reduced pressure is decisive in these synthesis reactions to remove the ethanol or water formed and to shift the reaction equilibrium toward high conversions. The lower pressures (4 mbar) used with free fatty acids by other authors could provide enhanced conversions into 1,3-diacylglycerols. Regarding this fact, Rosu et al. (1999) reported the use of molecular sieves, instead of vacuum, to adsorb the water formed when using butyric and caproic acid as the most volatile acyl donors, in order to prevent its elimination from the reaction medium. However, in our work, molecular sieves would be ineffective to remove ethanol, so that a controlled reduced pressure was needed.

**Figure 5:**
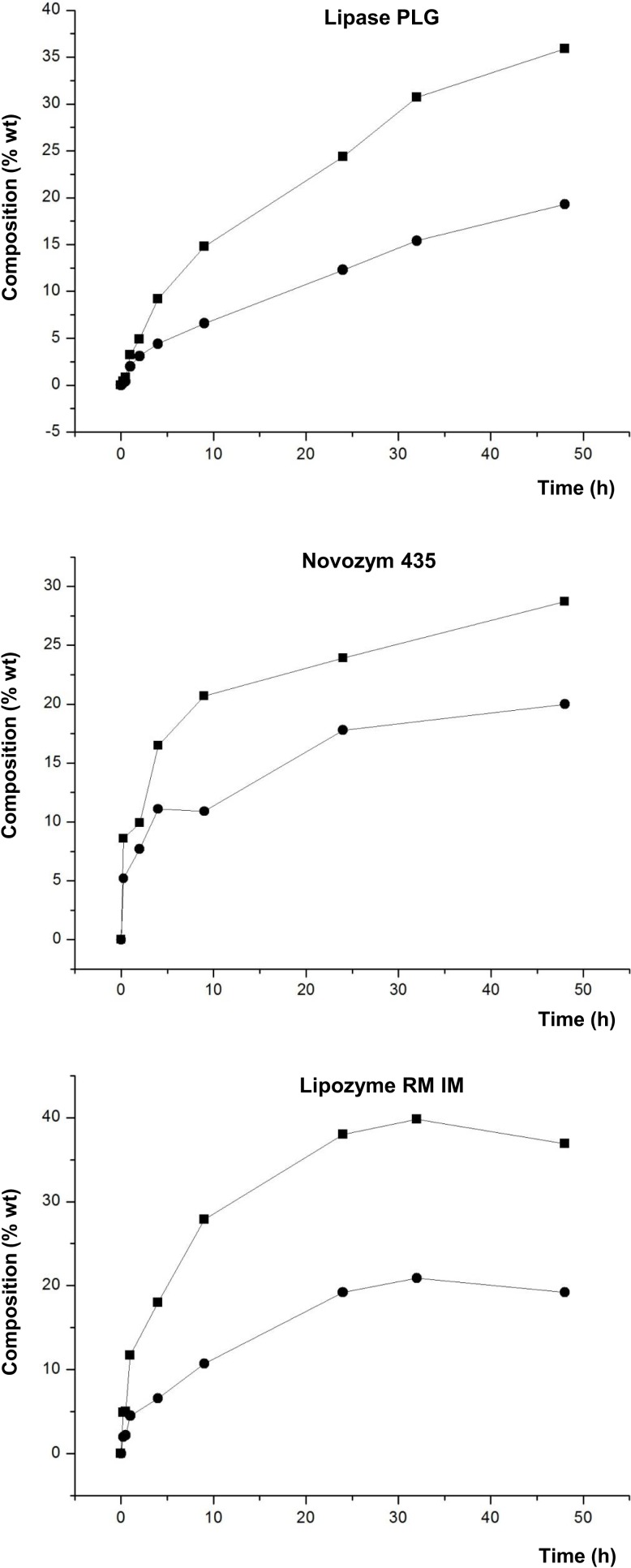
**Composition (% w/w) of 1,3-DAG (■) and 1,2-DAG (●) at different reaction times of transesterification between FAEE and glycerol adsorbed on silica gel with Lipase PLG (*Alcaligenes* sp.), Novozym 435 (*Candida antarctica*), and Lipozyme RM IM (*Rhizomucor miehei*)**.

Another point to be noted from the results of this study are the differences in the synthesis of 1,3-DAG and 1,2-DAG. Figure 5 shows the different proportions of these positional isomers of the diacylglycerol molecule.

Figure 5 shows that Lipase PLG and Lipozyme RM IM have similar behavior regarding composition of 1,3-DAG and 1,2-DAG at the optimum. Thus, with Lipase PLG, the amount of 1,3-DAG and 1,2-DAG was 35.9 and 19.3%, respectively, whereas with Lipozyme RM IM, it was 39.8% (1,3-DAG) and 20.9% (1,2-DAG). For both lipases, the relationship between 1,3-DAG and 1,2-DAG was approximately double. In contrast to these results, when Novozym 435 was used as a biocatalyst, the amount of these compounds was different, with 32.1% for 1,3-DAG and 19.9% for 1,2-DAG.

A reason to explain these results is the positional specificity of Lipase PLG and Lipozyme RM IM, preferably to esterify acyl groups in the *sn*-1 and *sn*-3 positions of the glycerol molecule. In contrast, Novozym 435 has no positional specificity. Because of these characteristics, it would be expected an increase in the synthesis of 1,2-DAG and also TAG with Novozym 435. However, it was observed that the proportion of TAG was similar in these three lipases. Lower TAG formation can be also related to the utilization of a moderate vacuum of 200 mbar, which is not high enough for TAG synthesis.

### Lipase-Catalyzed Reactions to Synthesize DAG Using Lecithin as Emulsifier

**Figure 6:**
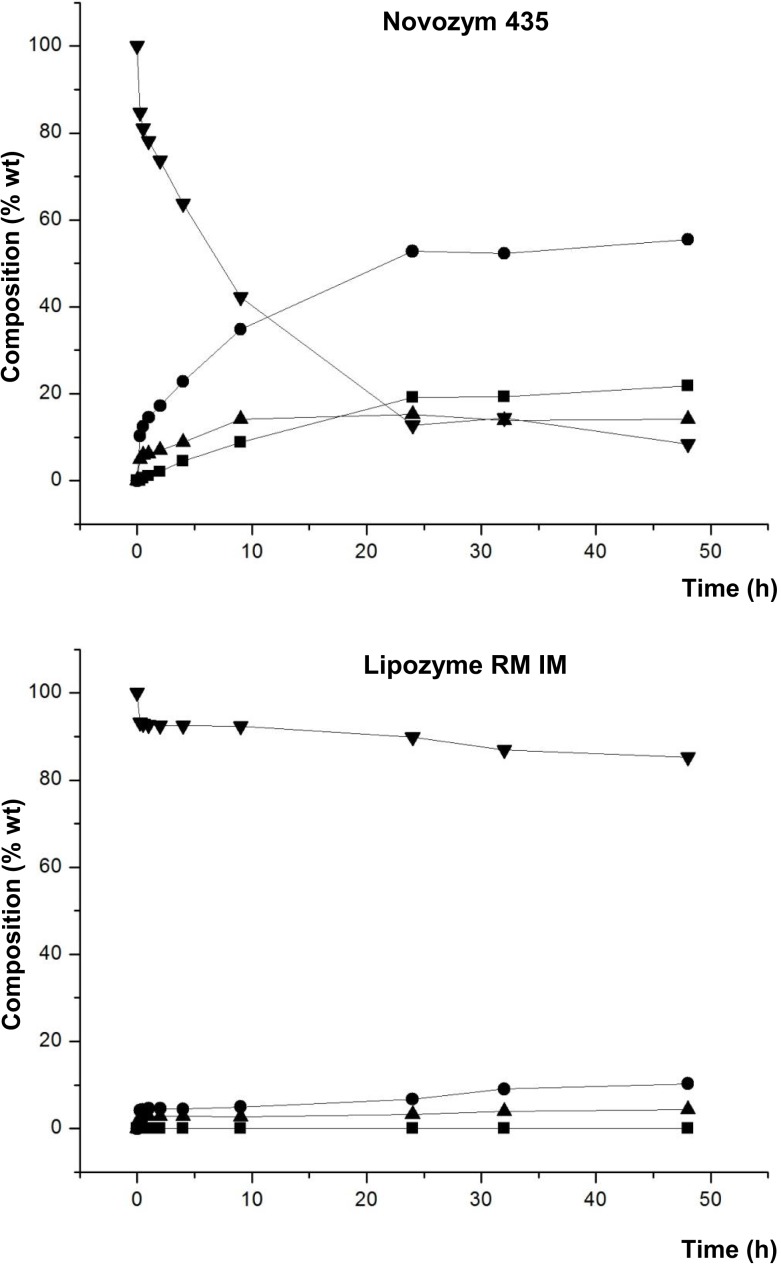
**Composition (% w/w) of FAEE (▼), TAG (■), DAG (●), and MAG (▲) at different reaction times of transesterification between FAEE and raw glycerol with Novozym 435 (*Candida antarctica*) and Lipozyme RM IM (*Rhizomucor miehei*), and adding 1% w/w lecithin**.

Finally, the effect of adding a small amount of lecithin (1% w/w) in the reaction medium was studied. Because of the emulsifying properties of lecithin, the objective was to improve the dispersibility of glycerol and FAEE, minimizing a possible coating of the active site of the lipase by glycerol, when it was not adsorbed on silica. This effect was studied using those lipases that provide better results of conversion of DAG and reaction rate, Novozym 435 and Lipozyme RM IM. Figure 6 shows the composition of the lipid phase without glycerol at the different reaction times.

In the presence of Novozym 435, remarkable differences were observed at these conditions in comparison with glycerol adsorbed on silica gel with no addition of lecithin (see Figure 4). Thus, both DAG synthesis and the reaction rate were significantly higher with raw glycerol and 1% lecithin. For example, after 24 h, DAG conversion increased from 41.7 to 52.8% DAG by using raw glycerol and 1% lecithin. Besides, comparing the results with raw glycerol, the addition of 1% lecithin provided higher proportion of glycerides, e.g., 52.8% DAG and 19.2% TAG (1% lecithin) vs. 47.7% DAG and 11.8% TAG (without lecithin), attained at 24 h (see Figure 3). Thus, an improved dispersion of the substrates by the lecithin could increase the reaction rate and promote a more favorable introduction of acyl groups at the secondary hydroxyl position of the glycerol molecule, resulting in an increased synthesis of TAG.

In contrast, in the reaction with Lipozyme RM IM, the addition of lecithin caused no improvement in the DAG synthesis, possibly due to the improved dispersion of glycerol did not prevent the coating of the lipase.

**Figure 7:**
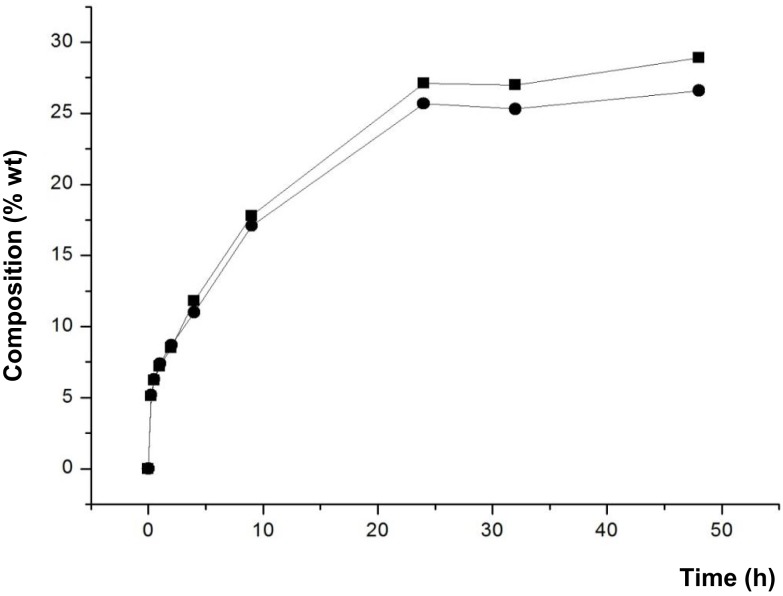
**Composition (% w/w) of 1,3-DAG (■) and 1,2-DAG (●) at different reaction times of transesterification between FAEE and glycerol adsorbed on silica gel with Novozym 435 (*Candida antarctica*), and adding 1% w/w lecithin**.

Furthermore, Figure 7 shows that the amounts of 1,3-DAG and 1,2-DAG synthesized were similar along the entire reaction when Novozym 435 was used with lecithin, in contrast to the ratio observed with glycerol adsorbed on silica gel without lecithin. As mentioned above, lecithin may enhance the homogenization of the reaction medium and it could change the orientation of the substrates at the interface, leading to an increased synthesis of 1,2-DAG.

Finally, the excess of FAEE present in the final product mixture was removed by distillation and the residual glycerol content was measured. The glycerol content was below 5% in all cases. Some replicates were performed at optimal conditions in order to confirm the reproducibility of the results. It was observed that the variation among the different assays was <5%.

## Conclusion

In this work, solvent-free synthesis of short- and medium-chain DAG has been achieved. Only Novozym 435 formed acylglycerols with raw glycerol. Other lipases required previous adsorption of glycerol on silica gel to prevent coating of the active site of the lipase by glycerol. The mentioned coating can be related to the chemical nature of the enzyme carrier and for this reason utilization of Novozyme 435 may be an advantage for a possible scale-up of the process.

Two optimal conditions to produce short- and medium-chain DAG were achieved:

- By using Novozym 435 (*C. antarctica*), raw glycerol and 1% of lecithin, 52.8% DAG was reached at 24 h of reaction. This process resulted in a product with a similar ratio of 1,3-DAG (27.1%) and 1,2-DAG (25.7%).
- By using Lipozyme RM IM (*R. miehei*) and glycerol adsorbed on silica gel, 60.7% DAG was reached at 32 h of reaction. This process resulted in a product with a higher content of 1,3-DAG (39.8%), compared to 1,2-DAG (20.9%).

The technology used is adequate for the production of hypocaloric food ingredients with functional properties and offer advantages regarding conventional methods, mainly due to the greener chemistry involved, selectivity, and efficiency. The highly valuable lipid ingredients obtained may be used in nutraceuticals and functional food production.

## Author Contributions

NG carried out the experimental research at laboratory. NG performed all the enzymatic reactions. NG also carried out the GC and HPLC analyses and the immobilization of glycerol on silica gel. LV participated in the HPLC and GC analyses and optimized these methodologies. LV planned the design of experiments and studied the different conditions to be evaluated. LV managed all the experimental procedures. LV also provided the starting material, consisting of short and medium FAEE obtained from molecular distillation of fatty acid ethyl esters of coconut oil and dairy fat. LV elaborated the final manuscript. CT collaborated in the design of experiments, the discussion of the results, and elaborating the final manuscript. GR supervised the work and managed the financial support to develop this project.

## Conflict of Interest Statement

The authors declare that the research was conducted in the absence of any commercial or financial relationships that could be construed as a potential conflict of interest.
